# Supplementary material for: Contribution of metabolomics to the taxonomy and systematics of octocorals from the Tropical Eastern Pacific
Source: PeerJ. 2025 Mar 12;13:e19009. doi: 10.7717/peerj.19009 (PMC11910152; doi:10.7717/peerj.19009)
Supplement: Supplemental Information 1 [file peerj-13-19009-s001.docx]

**Supplementary Information**

**Contribution of metabolomics to the taxonomy and systematics of octocorals from the Tropical Eastern Pacific.**

Karla B. Jaramillo^1, 2, 3, 4 *^, Paul O. Guillen ^1, 4, 5^, Rubén Abad ^4^, Jenny Antonia Rodriguez León ^3, 4^, and Grace McCormack.^2^

^1^ Marine Biodiscovery, School of Chemistry and Ryan Institute, National University of Ireland Galway, Galway, Ireland.

^2^ Zoology, School of Natural Sciences and Ryan Institute, National University of Ireland Galway, Galway, Ireland.

^3^ Facultad de Ciencias de la Vida, Escuela Superior Politécnica del Litoral, Guayaquil, Ecuador.

^4^ Centro Nacional de Acuicultura e Investigaciones Marinas, CENAIM. Escuela Superior Politécnica del Litoral, Guayaquil, Ecuador.

^5^ Facultad de Ciencias Naturales y Matemáticas, Escuela Superior Politécnica del Litoral, Guayaquil, Ecuador.

* Corresponding Author:

Karla B. Jaramillo^1, 2, 3, 4*^

Campus Gustavo Galindo Km. 30.5 Vía Perimetral, Guayaquil, Guayas, 09-01-5863, Ecuador.

Email address: [kbjarami@espol.edu.ec](mailto:kbjarami@espol.edu.ec)

*
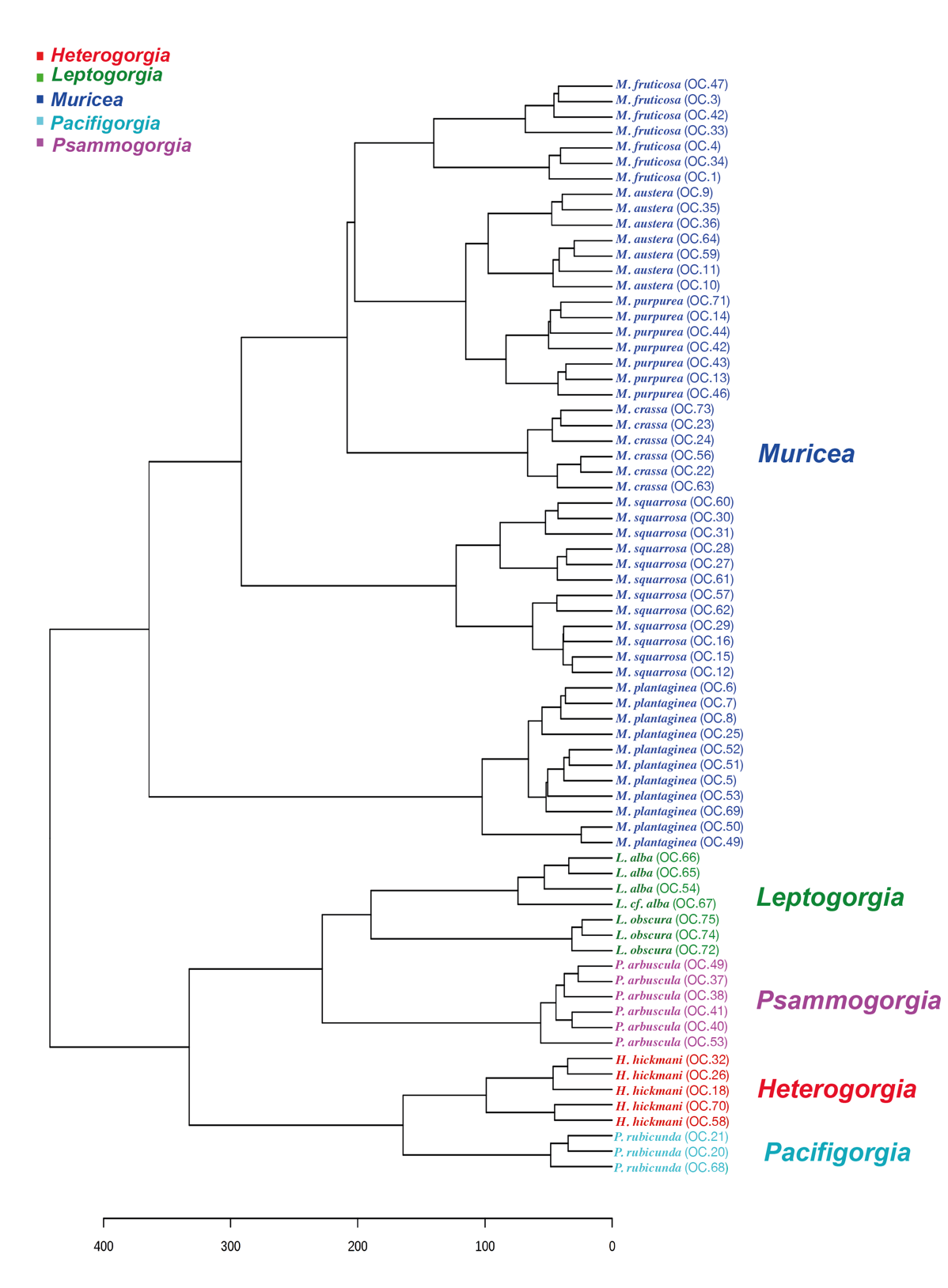
*

**Figure S1. Untargeted metabolomic analyses at genus level by Hierarchical Clustering Analyses (HCA).** Clustering result shown as dendrogram between the 71 metabolomic profiles of the five octocoral genera; *Heterogorgia* (red), *Leptogorgia* (green), *Muricea* (dark blue), *Pacifigorgia* (light blue), *Psammogorgia* (pink). (Distance measure using euclidean, and clustering algorithm using ward.D.).

*
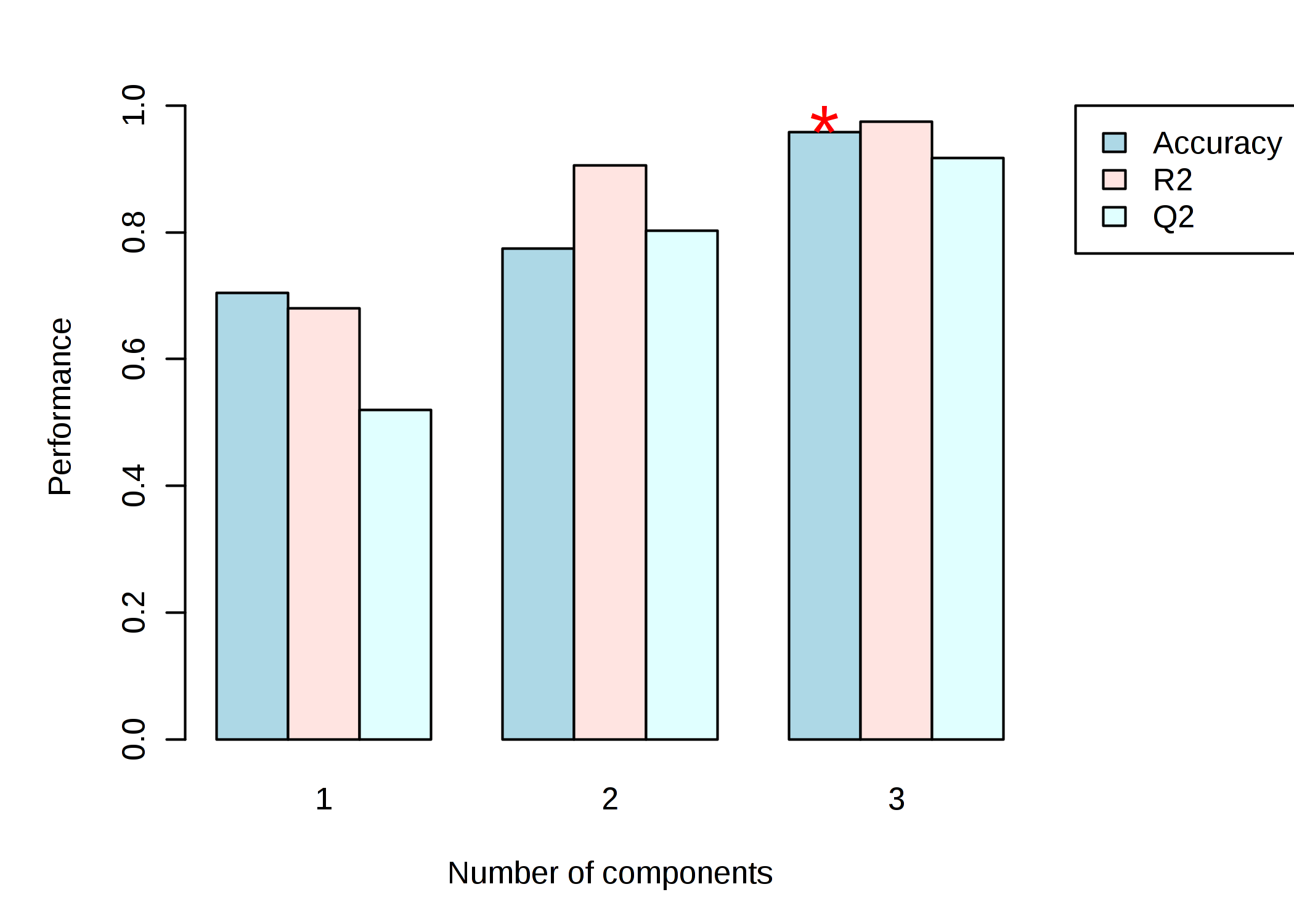
*

**Figure S2. Cross validation analyses at genus level.** Cross validation values of the 71 octocoral specimens of five genera using the classification performance of three principal components of the PLS-DA analyses at genus level. The red start indicates the best classifier.

| **PLS-DA parameters** | | | | |
| --- | --- | --- | --- | --- |
| **Chemical features** | **Q2** | **R2** | **Q2/R2** |  |
| PC1 | 0,51 | 0,67 | 0,70 |  |
| PC 2 | 0,82 | 0,88 | 0,73 |  |
| PC 3 | **0,92** | **0,99** | **0,97** |  |

**Table S1.** Parameters and permutation test for distinguish the five genera from 71 extracts of octocorals using the classification performance of three principal components of the PLS-DA (Partial least squares-discriminant analyses) analyses at genus level. Parameters in black color based on Q2 indicates the best classifier.


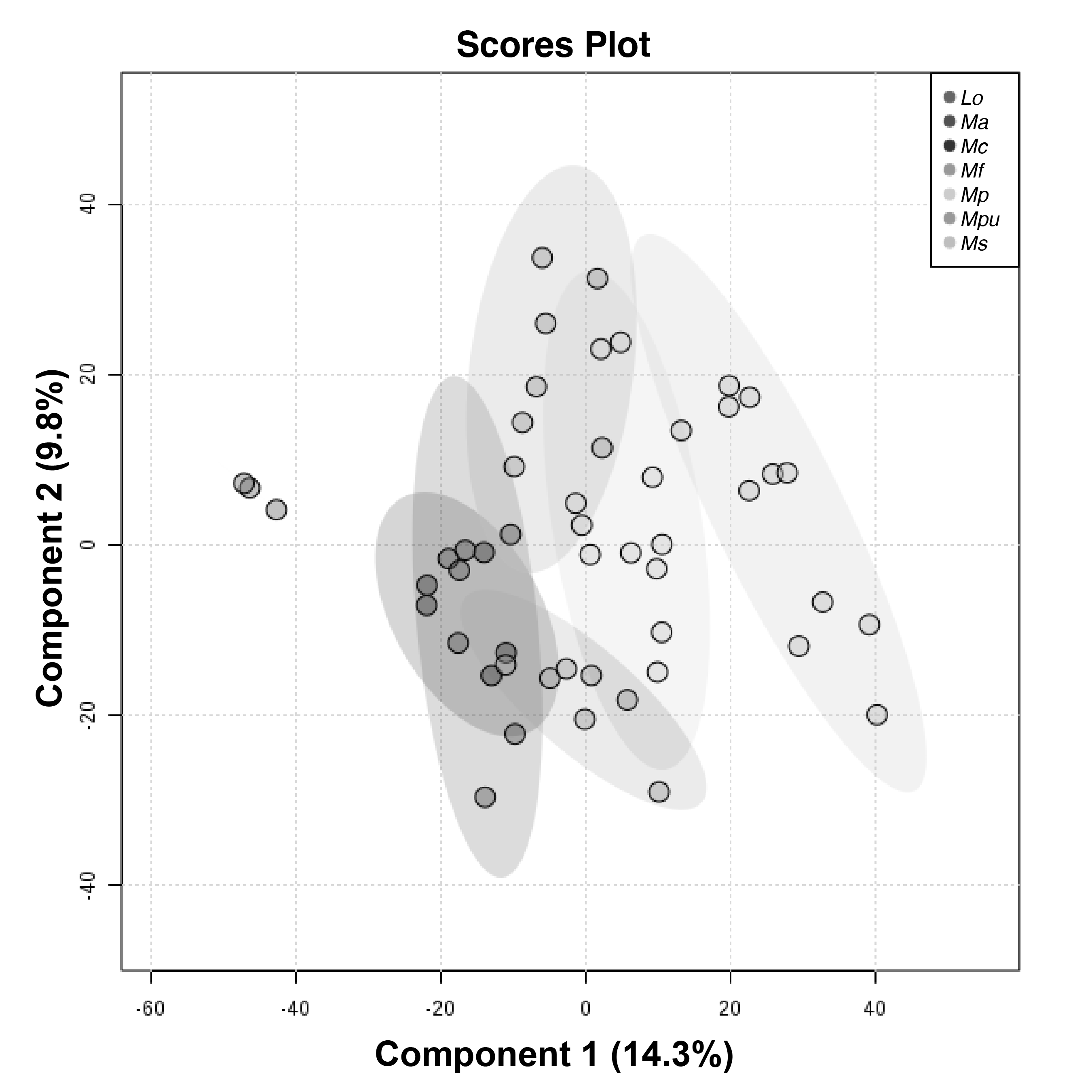


**Figure S3.** **Untargeted metabolomic at species level.** PLS-DA supervised analysis for *Muricea* species; *M. austera* (Ma, green), *M. crassa* (Mc, dark blue), *M. fruticosa* (Mf, light blue), *M. plantaginea* (Mp, pink), *M. purpurea* (Mpu, yellow), *M. squarrosa* (Ms, grey) and *Leptogorgia obscura* (Lo, pink) as an outgroup. Scores plot between the metabolomic profiles of the six *Muricea* species. The variances are shown in brackets and ellipses show a 95% of confidence.


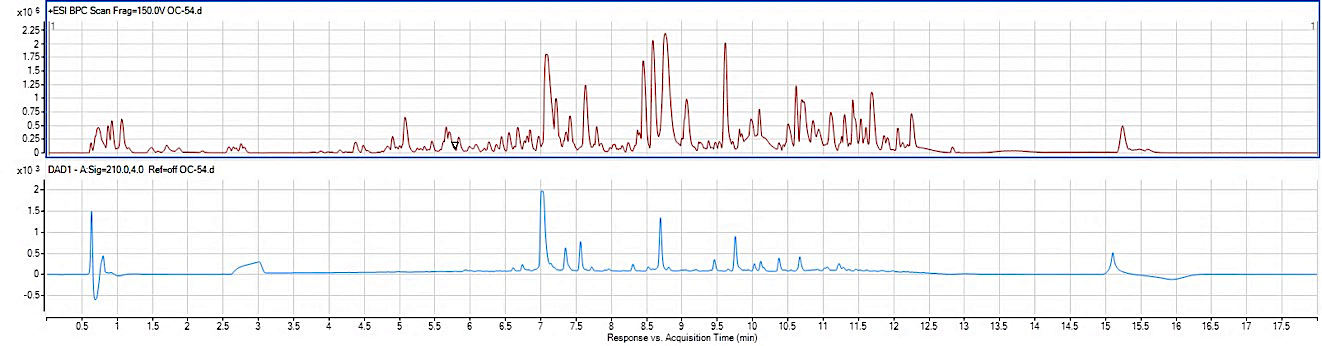


**Figure S4.** Metabolomic profile of *Leptogorgia alba* (OC-54) reported in this study.


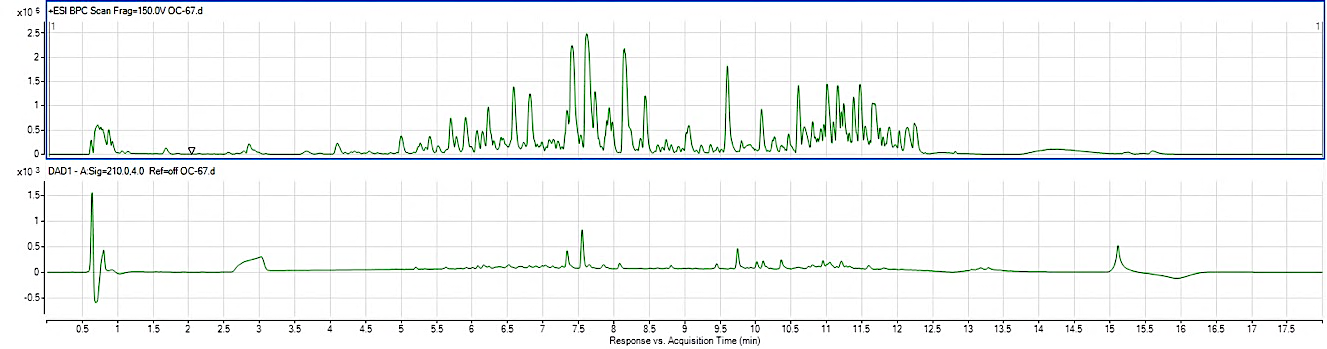
 **Figure S5.** Metabolomic profile of *Leptogorgia* cf. *alba* (OC-67) reported in this study*.*


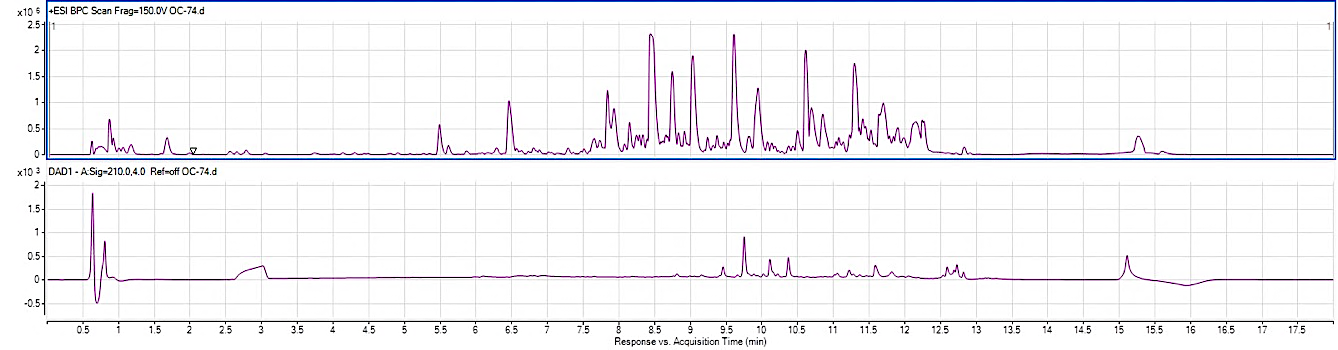
 **Figure S6.** Metabolomic profile of *Leptogorgia obscura* (OC-74) reported in this study*.*


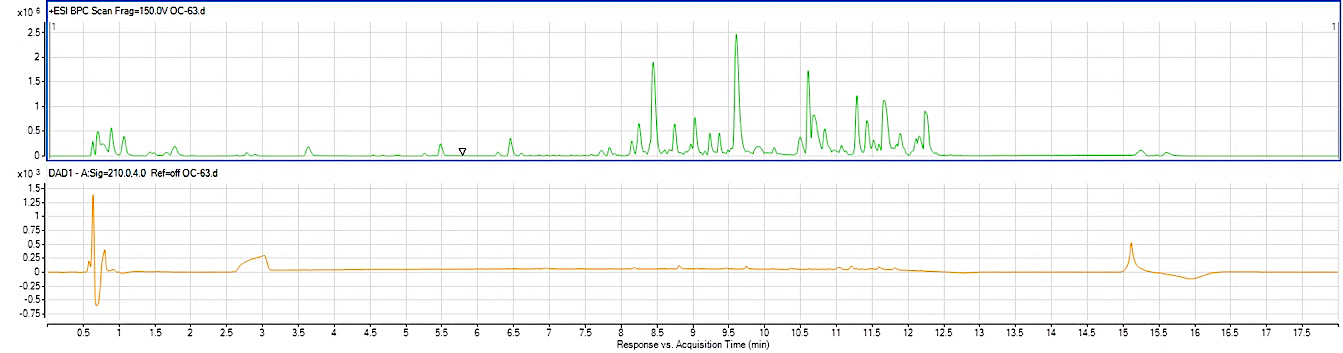
 **Figure S7.** Metabolomic profile of *Muricea crassa* (OC-63) reported in this study*.*


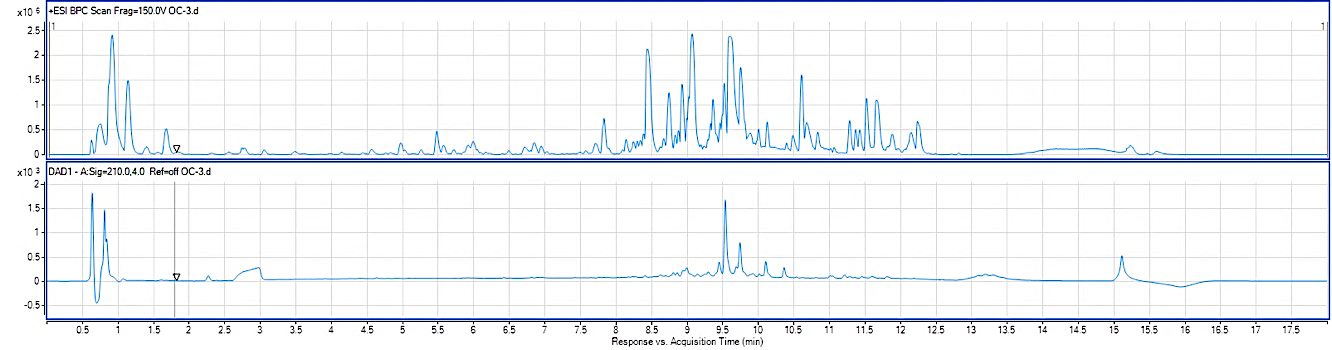
 **Figure S8.** Metabolomic profile of *Muricea fruticosa* (OC-3) reported in this study.


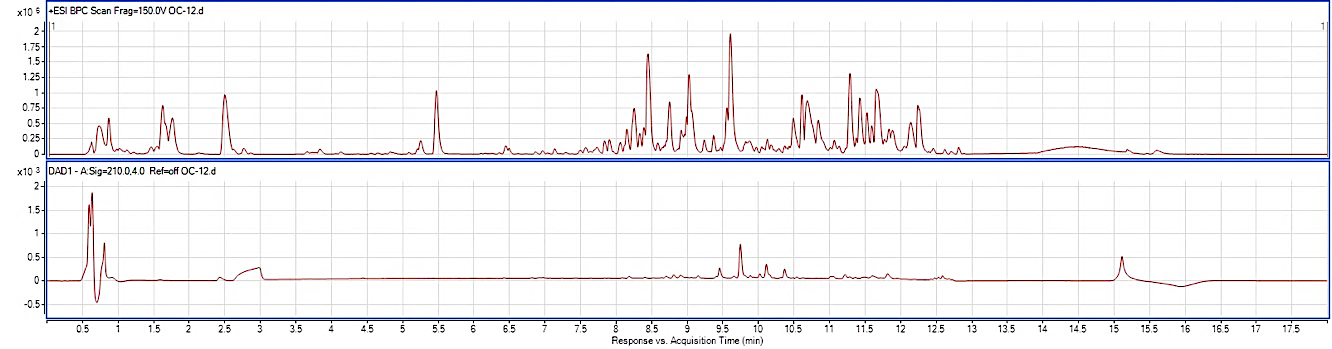
 **Figure S9.** Metabolomic profile of *Muricea squarrosa* (OC-12) reported in this study.


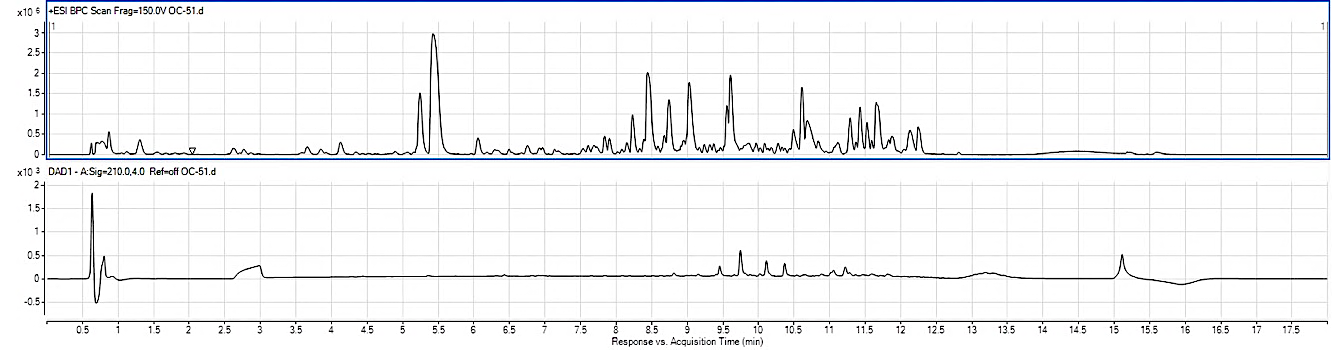
 **Figure S10.** Metabolomic profile of *Muricea plantaginea* (OC-51) reported in this study.


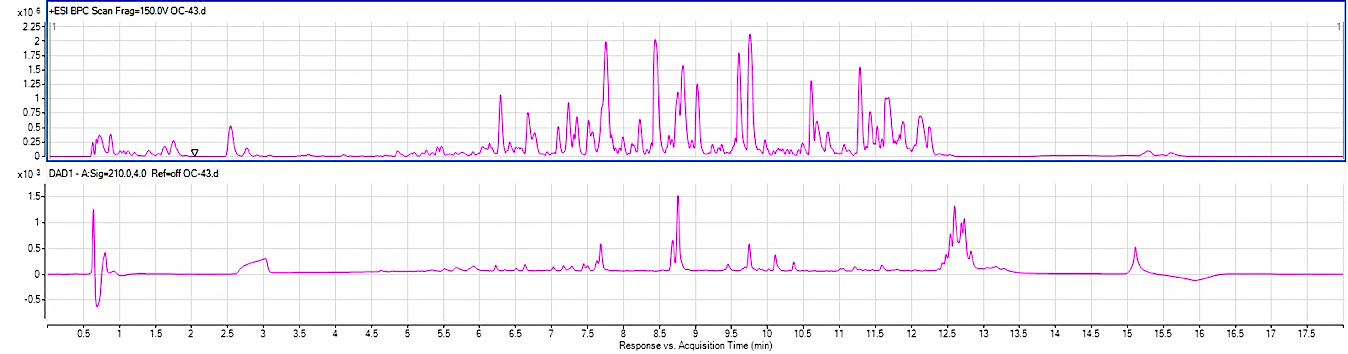
 **Figure S11.** Metabolomic profile of *Muricea purpurea* (OC-43) reported in this study.


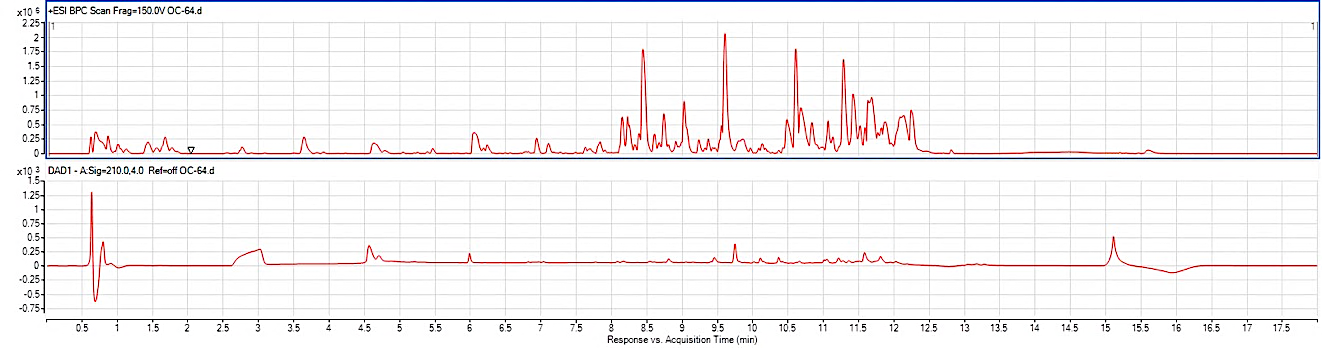


**Figure S12.** Metabolomic profile of *Muricea austera* (OC-64) reported in this study.


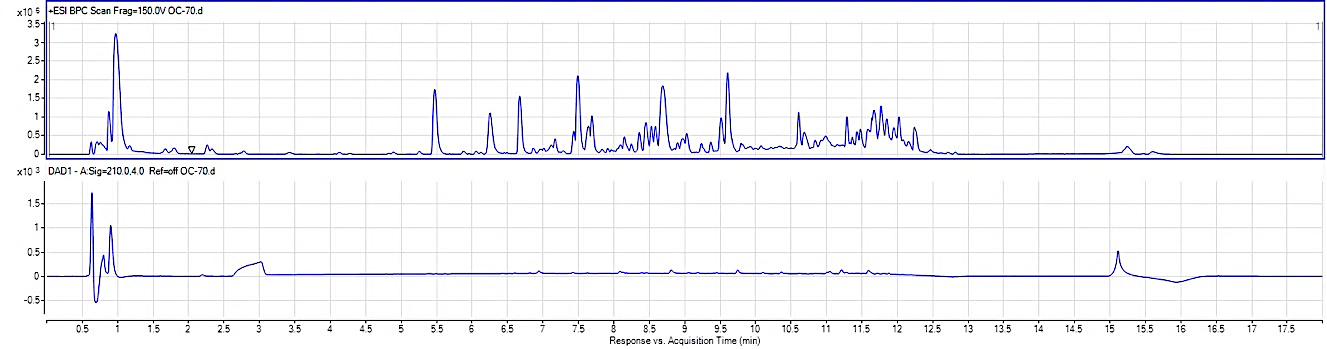
 **Figure S13.** Metabolomic profile of *Heterogorgia hickmani* (OC-70) reported in this study.


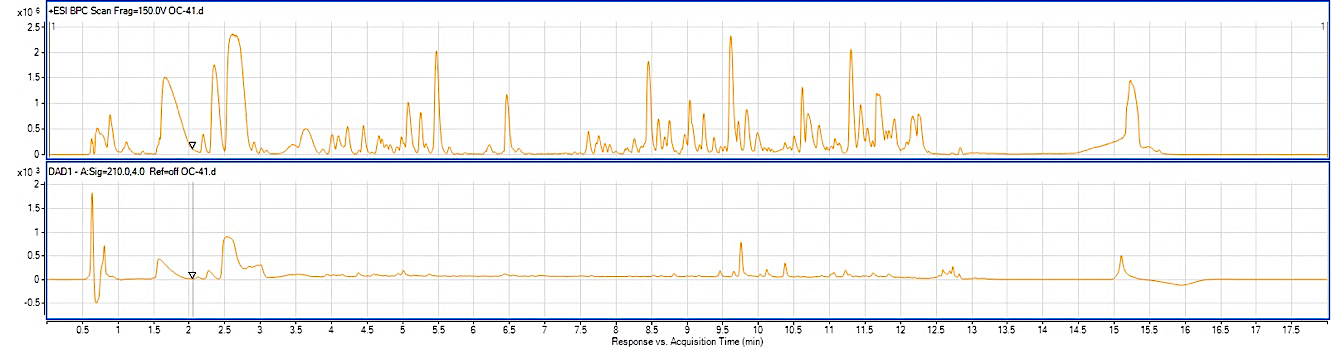
 **Figure S14.** Metabolomic profile of *Psammogorgia arbuscula* (OC-41) reported in this study.


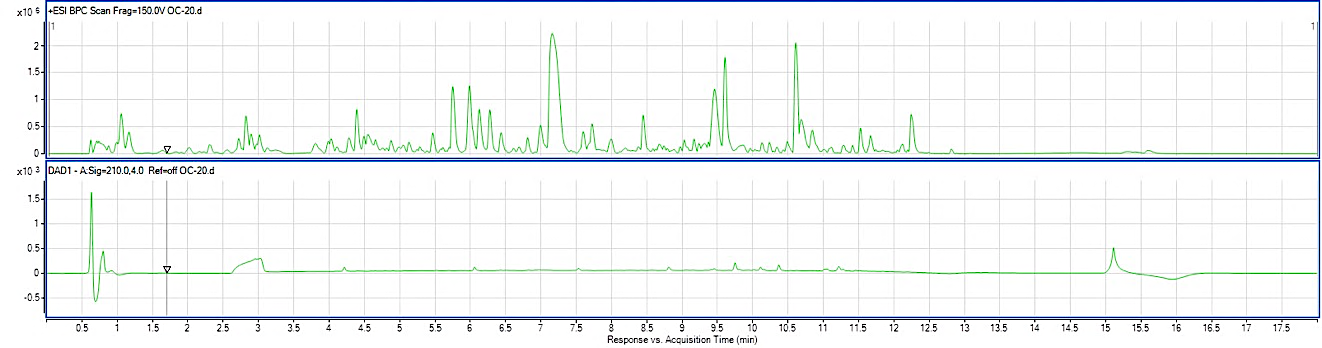
 **Figure S15.** Metabolomic profile of *Pacifigorgia rubicunda* (OC-20) reported in this study.
